# Germline Structural Variations in Cancer Predisposition Genes

**DOI:** 10.3389/fgene.2021.634217

**Published:** 2021-04-14

**Authors:** Tímea Pócza, Vince Kornél Grolmusz, János Papp, Henriett Butz, Attila Patócs, Anikó Bozsik

**Affiliations:** ^1^Department of Molecular Genetics, National Institute of Oncology, Budapest, Hungary; ^2^Hereditary Cancers Research Group, Hungarian Academy of Sciences, Semmelweis University, Budapest, Hungary; ^3^Department of Laboratory Medicine, Semmelweis University, Budapest, Hungary

**Keywords:** germline mutation, structural variations, cancer–predisposing genes, copy number variation, large genomic rearrangement, structural variation

## Abstract

In addition to single nucleotide variations and small-scale indels, structural variations (SVs) also contribute to the genetic diversity of the genome. SVs, such as deletions, duplications, amplifications, or inversions may also affect coding regions of cancer-predisposing genes. These rearrangements may abrogate the open reading frame of these genes or adversely affect their expression and may thus act as germline mutations in hereditary cancer syndromes. With the capacity of disrupting the function of tumor suppressors, structural variations confer an increased risk of cancer and account for a remarkable fraction of heritability. The development of sequencing techniques enables the discovery of a constantly growing number of SVs of various types in cancer predisposition genes (CPGs). Here, we provide a comprehensive review of the landscape of germline SV types, detection methods, pathomechanisms, and frequency in CPGs, focusing on the two most common cancer syndromes: hereditary breast- and ovarian cancer and gastrointestinal cancers. Current knowledge about the possible molecular mechanisms driving to SVs is also summarized.

## Introduction

The genetic diversity of the human genome is based on several types of variations from single nucleotide polymorphisms to large genomic rearrangements (LGRs). Chromosomal rearrangements comprise various types of structural variations (SVs). Some of them are copy-neutral (balanced) rearrangements, such as inversions and translocations, while others modify the dosage of chromosomal regions. These latter groups consist of copy number variations (CNVs), which are gains or losses of DNA fragments (deletions, duplications, or amplifications) constituting approximately 5% of the genome and providing the major source of genetic diversity (Zhang et al., 2009). Although CNVs may involve larger chromatin structures, the majority of them are subtle alterations of submicroscopic size generally ranging from 100 bp to 3 Mb (Zhang et al., 2009).

As opposed to recurrent genomic rearrangements, which are mainly gross recombinational events between chromosomal arms encompassing the same genomic interval in unrelated individuals, SVs in cancer predisposition genes (CPGs) are mainly non-recurrent events. This means that there is no particular genomic region for the breakpoints; these can arise at any chromosomal position. Only slight hot spots or grouping of breakpoints can be discerned with especially complex chromatin architectural structures or within pseudogene regions (Puget et al., 2002).

Structural variations appear either as somatic variations, especially in tumors, or can be generated in the germline. In this case, they are heritable. When SVs fall in functionally relevant regions of the genome, especially when they alter the open reading frame of coding genes, they seriously compromise gene function. This is especially remarkable in CPGs, where these changes contribute to the hereditary mutation profile and confer an increased risk for cancer. Noteworthy, SVs can also affect non-coding genes involved in cancer susceptibility (lncRNAs, microRNAs, and other types of small RNAs), some of which also have exon/intron structures.

Cancer predisposition genes are mainly tumor suppressors, which generally act recessively: both alleles should be lost for developing a phenotype. The Knudson two-hit model sets out that the first hit is an inherited germline mutation, which is followed by a subsequent somatically acquired second hit for tumor generation (Knudson, 1971). The second hit for tumorigenesis frequently appears in one individual’s lifetime, causing dominantly appearing cancer disease phenotypes.

The pathogenicity of the SVs is not directly obvious in all cases. While the majority are clear-cut mutations, there are cases, especially in certain duplications and inversions, where additional functional tests are required to assess their effect on clinical outcomes.

Continuously evolving sequencing technologies enable the detection of various types of SVs, which were formerly missed by conventional detection techniques. This allows the identification of an emerging number of rearrangements, which further broadens the spectrum of these variations.

Here, we provide a comprehensive review of germline SV types, detection methods, pathogenic mechanisms, and frequency in CPGs, focusing on three common cancer syndromes. These are hereditary breast- and ovarian cancer (HBOC) and two types of hereditary gastrointestinal cancers, i.e., familial adenomatous polyposis (FAP) and hereditary non-polyposis colorectal cancer (HNPCC or Lynch syndrome). Our work also covers rare hereditary cancer syndromes, each possessing a strong heritability factor and associated with acknowledged tumor suppressor genes.

## SVs – Underlying Causative Molecular Mechanisms

The molecular mechanisms generating rearrangements may be replicational or recombinational events and are mostly related to repair processes.

**FIGURE 1:**
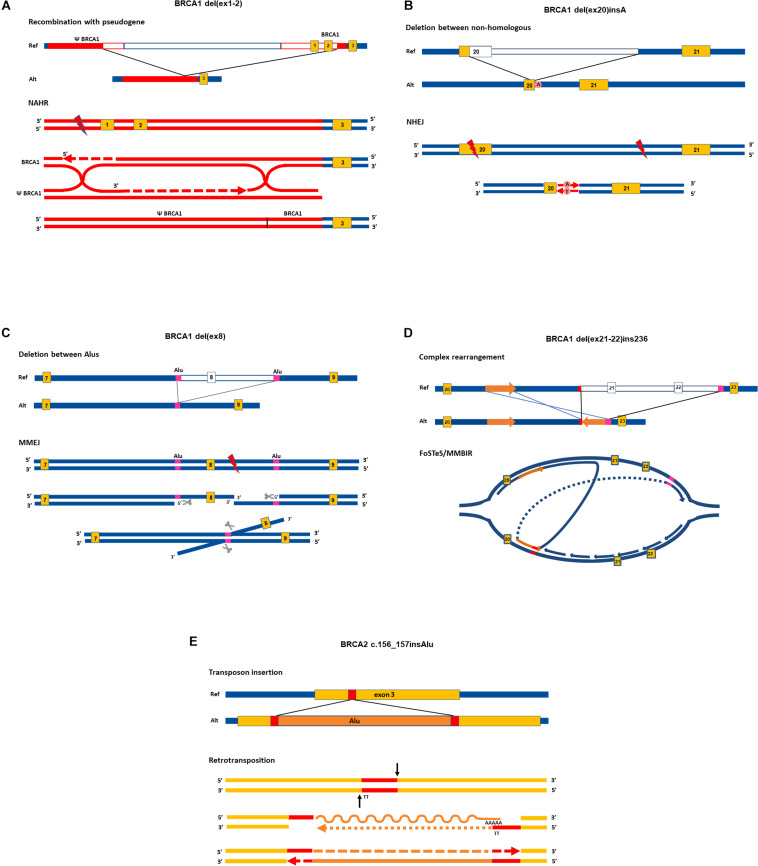
Proposed rearrangement types in cancer susceptibility genes. Upper parts of the panels show the allelic structure in sense orientation of elected rearrangements. Lower parts of the panels depict the most probable molecular mechanisms giving rise to the respective rearrangements, focusing on the main successive steps. Enzymes and auxiliary proteins mediating the mechanisms are not indicated. Yellow boxes indicate exons, red and magenta lines denote homologies, empty lines in reference allele mark deleted regions in alternative allele. Red lightning signs stand for double-strand break. Graphical representation of exons and introns is not to scale. The running name of the rearrangements are indicated above each graph, and exact names are given in the caption with HGVS nomenclature. All SVs taken as examples are registered variations in the LOVD database. NAHR, non-allelic homologous recombination, NHEJ, non-homologous end joining; MMEJ, microhomology-mediated end-joining; MMBIR, microhomology-mediated brake-induced repair; FoSTeS, fork stalling and template switching. Ref, reference allele; Alt, alternative allele. **(A)** NG_005905.2:g.61201_98134del. Running name: BRCA1 del(ex1-2) (Puget et al., 2002). At the position of the DNA double-strand break, the 5′ ends are resected and one of the overhanging 3′ ends invades into the D-loop of the homolog *Psi-BRCA1* region annealing with its complementary strand. Synthesis proceeds further for hundreds of base pairs, harnessing the ectopic homology as a template. Extensive homology between BRCA1 and its pseudogene enables the formation of double Holliday junction. The resolution of the double cross with recombination event results in a hybrid region of *Psi-BRCA1* and *BRCA1*. Single-strand nicks are sealed by polymerase (dashed arrows) and ends are rejoined by ligase. **(B)** LRG_292t1:c.5213_5278-2753delinsA. Running name: BRCA1 del(ex20)insA (Belogianni et al., 2004; Bozsik et al., 2020). DNA stretch between the two double-strand breaks is deleted and the two exposed ends are rejoined by NHEJ without homology requirement. Error-prone polymerase seals the nick by editing the sequence with an additional adenine residue at the synapsis. **(C)** LRG_292t1:c.442-1102_547+252del. Running name: BRCA1 del(ex8) (Bozsik et al., 2020). DNA ends are processed at the site of the double-strand break: 5′ strands are resected by exonucleases (marked with scissors). The overhanging 3′ strands find 26 base pairs with exact microhomology between nearby 300-bp-long and almost completely homolog AluSx and AluSp sequences (marked with purple boxes). The two strands anneal by the microhomology, and the protruding 3′ strand is eliminated by flap trimming (incision is marked with scissors). The DNA ends are rejoined by ligase. **(D)** LRG_292t1:c.5278-492_5407-128delins236. Running name: BRCA1 del(ex21-22)ins236 (Zikan et al., 2008; Bozsik et al., 2020). Replication forks stalls at structural hindrance caused by palindrome sequence. The 3′ end of the newly synthesized strand disassembles and reanneals to the complementer strand of the same replication bubble with the help of small homology of few nucleotides (indicated with red line). The synthesis proceeds in the reverse direction for 236 base pairs (marked with an orange arrow) and reanneals to the original template using another stretch of microhomology (marked with magenta line) skipping the intervening region. **(E)** NG_012772.1:g.8686_8687insAlu. Running name: BRCA2 c.156-157insAlu (Peixoto et al., 2011). RNA sequence from the AluYa5 inserts into the exon three through target primed reverse transcription method. Endonuclease incises (black arrows) at the ends of the target site (marked with red) liberating 3′ end with TT nucleotides. This serves as a complementer template for the polyA tail of the AluYa5 RNA to anneal. Reverse transcription priming is provided by the free 3′ end of the gene. After reverse transcription (indicated by dotted arrow) gaps are filled by polymerase. The RNA strand is lysed and exchanged with DNA also by 3′→5′ synthesis action of polymerase (dashed arrow). Lygase seals the ends. At the end of the retrotransposition process, the Alu sequence is inserted into the exon with the flanking duplication of the target site.

Non-allelic homologous recombination (NAHR) has long been acknowledged as the principal molecular process for SV generation (Sen et al., 2006). A decade ago, novel mechanisms were also discovered as possible casual events. Below, we summarize the most relevant molecular mechanisms calling forth SVs and present them in Figure 1 through selected examples of already proved rearrangements.

Non-allelic homologous recombination happens between false homologous alleles. This phenomenon, which is called illegitimate recombination, is the main source of recurrent rearrangements. The mechanism usually takes place during meiosis and mitosis and requires an extensive chromosomal homology region of several kilobases similar to conventional recombination (Weckselblatt and Rudd, 2015). Segmental duplications or low-copy repeats throughout the genome serve as a target for ectopic (non-allelic) alignment of the chromosomal regions (Bailey et al., 2002). Additionally, the human genome contains several thousands of transposon-related remnants and may even be located on different chromosomes, which might cause such illegitimate recombinations (Arkhipova and Yushenova, 2019). NAHR can take place between homologous chromosomes, but it can also be intrachromosomal (between sister chromatids) or take place during an intrachromatidal event. NAHR between chromosomal arms calls forth duplications and deletions. Intrachromatidal recombinations between direct repeats result in deletion, whereas inverted repeats serve as a target for inversions. Recurrent rearrangement in CPGs through NAHR is feasible in the case of extensive homology served by long pseudogene regions. Smaller-scale rearrangements are more likely to happen as a result of repair events. Homologous recombinational repair, occurring mainly during the S phase of the cell cycle, can also mispair with ectopic regions with similar sequences and can generate deletion and duplication of chromosomal fragments through a recombination-like resolution of the Holliday junction (Hastings et al., 2009b; Figure 1A). Non-homologous end joining (NHEJ) is a more error-prone end-joining repair, generally requiring no homology (Zhao et al., 2020). This mechanism is proposed for deletions, where no, or only a few base pairs of homology are detectable at the junction points. The insertion of additional nucleotides at the junction point (so-called scars) is characteristic of this repair (Figure 1B).

The breakpoints of the majority of CNVs encompassing exons of CPGs fall in Alu repetitive sequences. This indicates that Alu elements have a substantial role in the generation of exon-scale chromosomal rearrangements. Novel findings argue that NAHR events require more extensive homology than the typical 300 bp of Alu sequences (Kowalczykowski, 2015). Instead, for rearrangements between small stretches of homologies microhomology-mediated end-joining (MMEJ) mechanism was suggested (McVey and Lee, 2008; Sfeir and Symington, 2015; Sinha et al., 2016). This is a special repair mechanism at double-strand breaks, which involves 5′ strand resection and annealing of the 3′ overhangs mediated by nearby/proximal small homologies of 5–50 bases (Tournier et al., 2004; Figure 1C).

The combination of chromosomal segments, which are sometimes even distantly positioned, can occur as a result of replication-based molecular mechanisms: Fork Stalling and Template Switching (FoSTeS) and Microhomology-mediated Break-Induced Repair (MMBIR) (Hastings et al., 2009a). Despite the difference in the molecular background, these processes are not distinguishable by the resulted product: both give rise to complex rearrangements. Both mechanisms are preceded by stalled replication forks: the DNA polymerase is stopped either by palindrome loops and structural hindrance (FoSTeS) or breaks in the template strand (MMBIR) (Hastings et al., 2009a). The 3′ end disengages and anneals to another replication fork through microhomology of only a few (<6 bp) bases. The new fork, though positioned adjacently, may be distant in the chromosome, or even can be located on another chromosome. The polymerase uses this new strand as a template and replicates a stretch of this region before the strand reanneals to the original fork. Moreover, the 3′ end invasion to new replication forks can be repeated several times between different chromosomal regions before reannealing, thus entailing multiple, distantly located fragments coming together in juxtapositions (Colnaghi et al., 2011). This mechanism can also give rise to deletions, duplications with misaligned homology, and even reversions when the leading strand anneals to the lagging strand. The typical FoSTeS/MMBIR-resulted rearrangement in CPGs is a characteristic pattern of some kilobase deletion combined with a short stretch of reverse oriented duplication of a neighboring intronic segment (Figure 1D).

Retrotransposition is also a way for copy number gain. The main mobile elements in the human genome are Long INterspersed Element-1 (L1), SINE-VNTR-Alu (SVA), and Alu, the copy number of which continuously expands with replicative copy-and-paste retrotransposition. The mechanism involves the reverse transcription of the RNA of these elements and insertion of the cDNA copy into a new genomic position with the help of a special endonuclease (Goodier, 2016; Figure 1E). L1 elements are the only autonomous transposons in the current human genome. Alu and SVA elements have no capacity for reverse transcription themselves but harness the enzymatic activity of L1 elements for moving (Hancks and Kazazian, 2016).

It is important to note, that the results of the different mechanisms are overlapping. Therefore, the underlying molecular event is not unequivocally identifiable by the inspection of the rearrangement pattern.

## Pathomechanisms of SVs in Cancer-Predisposing Genes

There is a wide array of mechanisms by which a CNV can abrogate cancer gene function. The most typical is the deletion of one or more exons coding for indispensable domains or structural elements of the protein. Moreover, out-of-frame deletions, generated at any position of the open reading frame, result in premature termination codon (PTC) on the transcript, which either codes for a truncated protein or is eliminated by nonsense-mediated decay. When the deletion affects the promoter region, the regulation of the gene expression may be compromised. For example, a 10 kb promoter deletion in the *APC* gene affecting promoter 1B reduces the expression of *APC-1B* (Yamaguchi et al., 2016). If the rearrangements on the chromosome are more extensive, the deletion may cover the whole coding gene.

Contrary to the effect of deletions, the pathogenic effect of duplications is not straightforward. Breakpoint characterizations are needed for the detection of their exact positions and orientations for interpreting their genetic consequences. Out-of-frame tandem duplications generate PTCs and interfere with protein function similarly to deletions. The duplication of in-frame exons, resulting in two tandem copies of certain protein regions, theoretically, does not necessarily cause a severe adverse effect on the protein, if domain positions and functions are not affected. The same applies to promoter duplications, which sometimes also involve the first coding exons of the gene: in this case, there is at least one correct copy of the whole gene and optimal choice between the two promoters can help to evade the generation of an altered transcript. For example, the tandem duplication of 357 kb upstream of the *BRCA1* gene, reaching up to *BRCA1* exons 1–19, was evaluated as a benign variation (Du et al., 2018).

A special form of gene silencing is transcriptional interference (read-through). As an example, *MSH2* silencing can occur due to the deletion of 3′ exons of the upstream *EPCAM* gene (Ligtenberg et al., 2009). The *EPCAM* deletion eliminates the transcription termination signal, thus the RNA polymerase goes further towards the neighboring *MSH2* gene, preventing the binding of transcription factors to the *MSH2* promoter, consequently hindering its transcriptional initiation. Furthermore, the long transcript usually ends up in a PTC, directing this fused RNA towards nonsense-mediated decay, resulting in a deletion effect on both *EPCAM* and *MSH2* (Kovacs et al., 2009).

Retroelement (RE) insertions are rare events of great consequence regarding the functional abrogation on CPGs. Insertion of a RE (L1, SVA, or Alu elements) into exons or intron regions of genes may cause exon skipping, exonization (Schmitz and Brosius, 2011), PTC generation, or transcriptional interference (Kaer et al., 2011). Aberrant splicing as a result of RE insertion into splice regulation regions was also described. A prominent example for this latter effect is the insertion of an Alu-like element in *MLH1* intron 7, which interferes via a canonical splice donor site, leading to complete disruption of mRNA splicing (Li et al., 2020).

## SV Detection Methods

Precise determination of SVs is not an easy task. Due to their heterogeneity, there is no one standard procedure that allows the correct identification of both deletions, insertion, and copy number alterations involving multiallelic loci. Generally, molecular biological methods providing quantitative differences can be used for the detection of SVs (Cantsilieris et al., 2013; Butz and Patocs, 2019). Based on the size of SVs, different assays are available. Two widely employed approaches in routine clinical practice are hybridization-based and PCR-based techniques.

Fluorescent in-situ hybridization (FISH) is typically used for the identification of large genomic alterations, such as gross chromosomal abnormalities, but current advances in the technique enable the detection of CNVs with sizes as small as 50 kb. Fluorescently labeled DNA probes complementary to the sequence of specific regions are hybridized to metaphase chromosomes or interphase nuclei (Bayani and Squire, 2004). A state-of-the-art version of FISH providing even better resolution (5–500 kb) is fiber-FISH, where probes are visualized on mechanically stretched chromosomes (Ceulemans et al., 2012). This technique is especially appropriate for determining complex CNVs. Applying different fluorescence dyes, multiple DNA targets can be tested simultaneously, allowing for whole-genome analysis (multi-color FISH) (Ceulemans et al., 2012).

Another hybridization-based method is Southern blotting. Recently, due to its highly labor-intensive workflow, radioisotope labeling, and the requirement of high quantity and quality DNA, it has been mostly replaced by other techniques.

Of these approaches, microarrays, which belong to high-throughput techniques, are used to analyze the expression, genotype, or copy number of multiple genes simultaneously. In germline testing, array-based genotyping platforms (i.e., single nucleotide polymorphism-SNP arrays) are applied (Zhang et al., 2009). SNP arrays covering the entire genome or selected genetic regions using disease-specific SNP panels are also employed. The principle is based on the hybridization of fluorescently labeled probes detecting each genotype. By virtue of the intensity of the fluorescence signal, hetero-, hemi-, and homozygous variants may be distinguished, so the presence of either deletions or insertions of SVs can be determined. Loss of heterozygosity can be demonstrated by a parallel evaluation of normal and somatic DNAs of the same patient.

Array comparative genome hybridization (array CGH) uses a small glass slide (chip) that contains thousands of probes specific for certain regions of the genome. Fragmented sample and reference DNA are labeled with different fluorescent dyes, combined, and hybridized to the DNA probes on the array slide. After detection of the two fluorescent signals, the results are given as the ratio of test DNA to reference DNA at each probe. Depending on the chosen platform’s design and probe density, the resolution of CGH can vary from whole chromosomes to a few kilobases in size (Davies et al., 2005). In clinical practice, this is the most frequently employed cytogenetic assay. It is applied for the analysis of LGRs as well as submicroscopic structural alterations with unclear clinical importance. There are several databases (Database of Chromosomal Imbalance and Phenotype in Humans using Ensemble Resources; DECIPHER (Firth et al., 2009); International Standards for Cytogenomic Arrays Consortium; ISCA Consortium (Riggs et al., 2013), which aid in the interpretation of results. In tumor genetics, CGH is useful for the detection of somatic changes, including tumor heterogeneity and somatic mosaicism.

Optical genome mapping, an accurate high-throughput assay originally designed for aiding contiguous genome assemblies, is also applicable for the identification of all classes of SVs in the human genome (involving balanced events). Ultra-long, linearized DNA molecules are fluorescently labeled and digested with a combination of restriction enzymes. The nicks at the cleavages are optically detected as fluorescent signal discontinuities, which give a characteristic high-resolution restriction pattern for the DNA sequence. Dedicated software can assemble DNA stretches according to pattern similarities and comparative analysis of the strands enables the detection of divergent regions >500 bp caused by SVs. Optical mapping offers a significantly higher resolution than karyotyping on a similar scale to fiber-FISH.

Copy number changes can also be detected based on relative quantitations by qPCR or QMPSF (Quantitative Multiplex PCR of Short Fluorescent fragments). In both cases, the quantity of the examined region is compared to that of control regions with surely two copies. With the qPCR analysis, deletions are readily detectable by the difference of Ct values, each unit of Ct corresponding to two copy differences (Schmittgen and Livak, 2008). Limitations for duplications, however, do exist since the detection of a 2:3 ratio is not feasible. In contrast, QMPSF, where the area under the curve of the sample and control peaks yielded by multiplex PCR are compared, is amenable also for the detection of duplications (Ceulemans et al., 2012).

Inverse PCR is a suitable method for the verification of single inversions with known breakpoints. The principle of the detection is, that a PCR product is generated only when the primers hybridizing to the same strand in a reference template get into opposing orientations as a result of inversion (Wagner et al., 2002). New inversions may be discovered by allelic dropout test following long-range PCR. It is based on the phenomenon, that amplicons covering an inversion breakpoint appear as spurious deletions of one allele, presenting as stretches of homozygosity spanning the position of the inversion variant (Rhees et al., 2014). The detection of insertions is similarly demanding since their insertion point reside mainly in introns, which are not genotyped routinely. cDNA-level analysis of the genes may shed light on a part of these rearrangements since some of them generate new exons (exonization) (Schmitz and Brosius, 2011).

Multiplex ligation-dependent probe amplification (MLPA) is a semi-high-throughput technique developed to detect copy number alteration of up to 50 genomic DNA sequences in a single multiplex PCR-based mode (Kozlowski et al., 2008). Both internal control probes and positive-negative control samples have to be used during the analysis. First normalizing to internal controls (positions that are typically not affected by copy number alterations) in each sample, and then normalizing to control samples yield the relatively quantitative determination of the dosage in each probed locus. MLPA is an efficient way for detecting large deletions even in the hemizygote state. In a molecular genetic analysis of hereditary cancer syndromes, assays and complex reagents are available, and some of them have been already approved for in vitro diagnostic applications^1^. It has to be noted, that sequence polymorphisms within the ligation site can disturb the ligation sufficiently to cause a false positive deletion call (Serizawa et al., 2010).

Next-generation sequencing (NGS) is a high-throughput technology allowing simultaneous sequencing of multiple DNA samples. Currently, NGS-based procedures are the most widely used techniques in the routine molecular genetic diagnosis of hereditary cancer syndromes (Sarkadi et al., 2018). These approaches allow simultaneous determination of germline mutations and somatic alterations in sporadic tumors but have several requirements both from the sample and investigator sides (Robson et al., 2015). Complex laboratory workflow followed by bioinformatic analysis is needed for data mining (Krumm et al., 2012). Genotyping based on read depth analysis allows absolute copy number determination. Basically, the number of sequencing reads that map to a specific region is proportional to the number of copies of this region in the genome. The original hypothesis applies the Poisson distribution of sequencing reads, which means that a region assumed to be deleted or duplicated has fewer or more mapped reads than expected, respectively. Regarding instrumentation and sequencing chemistry, a wide selection of NGS analyses can be performed. In everyday practice, targeted gene panel sequencing (i.e., cancer panels, metabolic panels, pharmacogenetic panels, etc.) and whole exome sequencing are the most widely employed. Copy number determination from exome sequencing data is challenging because the coverage of coding exomes by sequencing reads is not uniform and can be biased by sequence capture design (Robson et al., 2015; Deans et al., 2017). Recent advances in computational approaches allow increasingly accurate determination of SVs and by unraveling the whole sequence, the correct breakpoints can also be determined (Oliver et al., 2015).

In summary, there are numerous methods available for the determination of SVs, but there remains no gold standard approach. Based on clinical practice, a combination of these techniques (i.e., array CGH and MLPA, NGS and MLPA, or qPCR) would allow the best diagnostic accuracy. The introduction of NGS technology and the development of computational data analysis will significantly increase the throughput and improve the accuracy of determining SVs.

## Examples of SVs in Cancer-Predisposing Genes

The size of germline deletions affecting cancer-predisposing genes ranges from few hundreds of base pairs to several kilobases (Bozsik et al., 2020). Chromosome regions characterized by abundant directly oriented repeats, especially Alu sequences, are markedly prone to deletions primarily through the MMEJ mechanism (Smith et al., 2016). Frequently occurring deletion types are single exon deletions and multi-exon deletions. A typical example for the former is *BRCA1* del(ex8) (Sluiter and van Rensburg, 2011; Bozsik et al., 2020; Figure 1B) and for the latter *CHEK2* del(ex9-10) (Cybulski et al., 2007), and both variants cause frameshifts at the transcript level. The deletion of the full *BRCA1* gene, del(ex1-24) has been previously detected in various populations (de la Hoya et al., 2006; Engert et al., 2008; Fachal et al., 2014). Pseudogene regions of a gene can serve as long sequence stretches with considerable homology for NAHR events, thus providing hot-spots for illegitimate recombinations that often result in deletions. There are several rearrangements with different breakpoints between *BRCA1* and its pseudogene (*Psi-BRCA1*), generating a ∼37 kb deletion involving the *BRCA1* promoter and exons 1–2 (Puget et al., 2002). Similarly, the *PMS2* locus also has multiple pseudogenes, especially *PMS2CL*, which has an almost 100% sequence identity with *PMS2* exons 12–15. This exact sequence homology enables dynamic gene conversions and recombinations between the two regions (Kohlmann and Gruber, 1993). Concerning genotype-phenotype correlations, there is no evidence for HBOC genes, *BRCA1* and *BRCA2*; whole exon deletions manifest in a more severe phenotype of the disease than smaller-scale indels (Gad et al., 2003; Walsh et al., 2006; Smith et al., 2016; Bozsik et al., 2020). Whole exon deletions in Lynch syndrome genes, *MLH1* and *MSH2*, however, are associated with a slightly earlier age of onset for colorectal cancer than small truncating variants, but this difference does not reach the nominal significance of 0.05 (Smith et al., 2016). In contrast, whole gene deletions may have altered phenotypic consequences in some syndromes: whole *NF1* deletions tend to cause a more severe phenotype, whereas whole *NF2* deletions generally result in a milder phenotype than truncating point mutations (Smith et al., 2016). Nevertheless, genetic alterations affecting additional causative genes may correlate with disease phenotype: in the case of a 7.4 Mb deletion encompassing *NF2* and neighboring genes corresponds to a more severe phenotype (Smith et al., 2016). In another example of contiguous gene deletion, germline 10q chromosomal deletion resulted in the loss of both *PTEN* and *BMPR1A*, and this corresponds to distinct pathological features of polyposis syndromes, underlining the complex interactions of these genes in tumorigenesis (Delnatte et al., 2006). Similarly, contiguous gene deletion within the 2p16-p21 chromosomal region, encompassing *MSH2, MSH6, EPCAM*, and 24 additional genes, causes Lynch syndrome with distinct phenotypic features (Salo-Mullen et al., 2018).

The majority of genomic duplications are directly oriented tandem repeats in CPGs (Sluiter and van Rensburg, 2011) and genome-wide (Newman et al., 2015) as well. The most prevalent tandem duplication in the *BRCA1* locus is *BRCA1* dup(ex13), which was detected in high frequency in nearly all European populations (The BEDSG, 2000). The bulk of single-exon or multi-exon duplications in CPGs reported so far are unambiguously pathogenic, although duplications encompassing the whole promoter together with a various number of downstream exons are evaluated as variants with unknown significance. For example, the examination of the *BRCA1* dup(ex1-2) variant by Fachal et al. (2014) failed to identify any aberrant transcripts (Fachal et al., 2014). Pathogenicity of other exon duplications detected by dosage-sensitive genotyping tests must also be confirmed by precise breakpoint assessment, as it was done for *BRCA2* dup(ex22-24) by van Luttikhuizen et al. (2020). They revealed, that the duplicated region was arranged in tandem and direct orientation, generating a PTC (van Luttikhuizen et al., 2020).

Particular types of copy gains include the insertion of mobile REs. Qian et al. (2017), conducted a large pan-cancer study on a panel of 26 genes and found that RE insertions were identifiable in 10 of the 26 genes tested (Qian et al., 2017). Indeed, RE insertions were detected in several genes (*BRCA1/2*, *APC*, *ATM*, *PMS2*, *MLH1*, and *MSH2*) by other groups studying hereditary breast and gastrointestinal cancers (Kaer et al., 2011; Li et al., 2020). Insertions of Alu repetitive motifs into exonic or intronic regions are the most prevalent transposition events in cancer-predisposing genes (Kaer et al., 2011). An Alu insertion in exon 3 of the *BRCA2* gene caused exon 3 skipping, and this is a founder mutation in the Portuguese population (Machado et al., 2007; Peixoto et al., 2011). In the Lynch syndrome-associated gene *PMS2*, insertion of an SVA nonautonomous retrotransposon element in intron 7 causes partial exonization of SVA using cryptic splice sites (van der Klift et al., 2012). *APC*, the germline susceptibility gene for FAP is disrupted by the insertion of an L1 sequence into exon 16 (Miki et al., 1992).

The detection of inversions can be challenging, therefore, their contribution to the SV pool is underestimated. In HNPCC, a 10 Mb paracentric inversion involving exons 1–7 of the *MSH2* gene was described first (Chen, 2008). This inversion was found to be a frequent cause of Lynch syndrome in a US population, accounting for an appreciable percentage of the mutational burden of this gene (Rhees et al., 2014). Later, another cryptic paracentric inversion of exons 2–6 of the same gene was detected (Liu et al., 2016). Germline inversion has also been shown for the *MLH1* locus, another major susceptibility gene in HNPCC. In this latter case, the inversion breakpoints are in intron 15 of *MLH1* and intron 3 of the neighboring *LRRFIP2* genes, generating two fusion transcripts between *MLH1* and *LRRFIP2* (Morak et al., 2011).

Translocations of whole chromosome arms are not typical events for disrupting tumor suppressor genes. However, two isolated cases with different chromosomal arm interchanges were described so far, each affecting the *APC* gene—a constitutional reciprocal translocation *t*(5;10) (van der Luijt et al., 1995) and a *t*(5;7) translocation (Sahnane et al., 2016).

The combination of rearrangement types manifests in complex genomic rearrangements. Despite these rearrangements possessing more than one junction point, they often arise from one molecular event, typically FoSTeS/MMBIR in cancer susceptibility genes. The characteristic pattern of deletion together with reverse duplication of some hundred base pairs occurs in various independent CNVs. *BRCA1* del(ex21-22) with reverse-oriented insertion of 236 bp of an intronic repeat is a founder complex CNV in the Czech population (Zikan et al., 2008; Ticha et al., 2010; Figure 1D and Table 1) and has also been reported as a recurrent variant in other European countries (Sluiter and van Rensburg, 2011; Bozsik et al., 2020). A complex recombination event characterized by the deletion of exons 5–10 and the insertion of a 35-bp nucleotide stretch in inverted orientation derived from the intron 3 sequence of the *BRCA1* gene is also a Czech founder mutation (Ticha et al., 2010). A deletion of exons 6–8 of *MLH1*, with the retention of 349-bp of intron 6 is also a complex rearrangement reported in one patient with colorectal cancer (McVety et al., 2005).

**TABLE 1:** Examples of founder SVs and frequencies in various populations.

Gene	SV (Running name)	Variant (HGVS)*	Population	Cancer syndrome	Frequency relative to gene mutations	Frequency in families of the syndrome	References
BRCA1	del(ex22)	NG_005905.2:g.168752_169261del	Dutch (Holland)	HBOC	36% of BRCA1(+)	NA	Petrij-Bosch et al., 1997
BRCA1	del(ex13)	NG_005905.2:g.133766_137600del					
BRCA1	del(ex3–16)	NC_000017:g.8655_55240del46586 NM_007294.3:c.81-1018_4986 +716del46586	Danish	HBOC	9/642 BRCA1/2(−)	NA	Hansen et al., 2009
BRCA1	del(ex17)	L78833:g.58530_61209delNG_005905. 2:g.147782_150460del	German	HBOC	NA	NA	Engert et al., 2008
BRCA1	del(ex5–14)	NG_005905.2:g.110966_142550del NM_007294.3:c.135-485_4485-913del31583	Czech	HBOC	NA	4/239	Ticha et al., 2010
BRCA1	del(ex1–17)	NM_007294.3:c.1-21434_5075-1084del80496					
BRCA1	del(ex21–22)	NG_005905.2:g.166375_170153delins:g. 162086_162321			NA	1/96, 2/172	Vasickova et al., 2007; Zikan et al., 2008
BRCA2	c.156_157insAlu	NG_012772.1:g.8686_8687insAlu	Portuguese	HBOC	NA	NA	Teugels et al., 2005; Machado et al., 2007
BRCA1	del(ex23–24)	NM_007294.3:c.5406+664_*8273del 11052L78833:g.80280_91331del NG_005905.2:g.169527_180579del	Greek	HBOC	22/181 BRCA1(+)	35/2092	Konstantopoulou et al., 2014; Apostolou et al., 2017
BRCA1	del(ex20)	NM_007294.3:c.5256_5277+3179del 3200L78833:g.71660_74860del3200			7/181 BRCA1(+)	7/760	
BRCA1	del(ex24)	NM_007294.3:c.5468-285_5592+4019del4429_insCACAGL 78833:g.82651_87079del4429_ins5			13/181 BRCA1(+)	13/720	Konstantopoulou et al., 2014
BRCA1	dup(ex13)	L78833:g.44369_50449dupNG_005905. 1:g.133622_139702dup	Northern British	HBOC	NA	NA	The BEDSG, 2000
BRCA1	del(ex9–12)	NG_005905.1:g.118955_133611del	Hispanic	HBOC	4/106 BRCA1/2(−)	NA	Weitzel et al., 2007
BRCA1	del(ex3–5)	L78833:g.8097_22733delNG_005905. 2:g.97346_111983del	Eastern Spanish	HBOC	10,97% of BRCA1(+)	NA	Palanca et al., 2013
CHEK2	del(ex9–10)	NM_007194.3:c.909-2028_1095+330del5395	Czech	HBOC	NA	NA	Cybulski et al., 2007
MLH1	del(ex17–19)	NM_000249.3:c.1896+280_oLRRFIP 2:c.1750-678del	Portuguese	HNPCC	17% of MMR(+)	NA	Pinhero et al., 2011
MSH2	del(ex7)	NM_000251.2:c.1077-3513_1276 +5655	Spanish	HNPCC	47% of MSH2(+)	7/160	Perez-Cabornero et al., 2011
MSH2	del(ex4–8)	NM_000251.2:c.646-1019_1386+2420del					
MSH2	del(ex1–6)	chr2:g.47,618,487_47,650,860delins (155); hg19	United States	HNPCC	NA	NA	van der Klift et al., 2005
APC	del(promB)	chr5:g.112,703,831-112,710,688; GRCh38/hg38	Italian	FAP	NA	NA	Marabelli et al., 2017

*Reference sequences and nomenclature for variants are taken from the articles cited. Running names are the short forms of the respective variants. SV, structural variation; HBOC, hereditary breast and ovarian syndrome; HNPCC, hereditary non-polyposis cancer syndrome; FAP, familial adenomatous polyposis; BRCA1(+), *BRCA1* mutation carriers; BRCA1/2(−), *BRCA1/2* mutation non-carriers; MMR(+), *MLH1*, *MSH2*, and *MSH6* mutation positives; MSH2(+), *MSH2* mutation carriers.

## Frequency of Germline SVs in Cancer-Predisposing Genes

The type and frequency of germline SVs in cancer susceptibility genes show wide differences across various populations. This is conceivably due in part to the different detection methods applied, the various and sometimes limited number of patients tested, as well as the founder alterations specific to certain populations. Founder variants are genetic alterations with a common origin, which are generated in an ancestor and spread through generations in an isolated ethnic group, thus these are recurrent and characteristic of a population. For example, haplotype analysis revealed, that the recurrent *BRCA1* deletion of exons 23 and 24 is a Greek founder mutation (Apostolou et al., 2017). Similarly, a recurrent exon 22 deletion in the *BRCA1* gene was found in the Netherlands (Sluiter and van Rensburg, 2011). Duplication of *BRCA1* exon 13 of Northern British origin (The BEDSG, 2000), as well as the above-mentioned deletion of exon 21-22 in *BRCA1* of Czech origin, are also frequently occurring CNVs in all populations in Europe (Ticha et al., 2010; Sluiter and van Rensburg, 2011; Bozsik et al., 2020). A large screening study in the US revealed, that 70.8% of all *BRCA1* rearrangements of Western and Northern European origin are made up of five founder CNVs (Judkins et al., 2012). Table 1 summarizes some examples of founder pathogenic SVs and their frequencies in the source populations.

Deletions are the most prevalent CNV types in cancer susceptibility genes (Sluiter and van Rensburg, 2011; Mancini-DiNardo et al., 2019; Bozsik et al., 2020), contributing to approximately 80–85% of all rearrangements (Mancini-DiNardo et al., 2019). In contrast, duplications account for only 10–15% of rearrangements (Mancini-DiNardo et al., 2019; Bozsik et al., 2020). The predominance of molecular processes resulting in genomic deletions compared to duplications might explain the observed difference between the frequencies of these alterations (Hastings et al., 2009b). RE insertions also represent a significant SV type as they accounted for one in every 325 unique pathogenic variants detected in a large pan-cancer study (Qian et al., 2017). In this cohort, 92% of all RE events were retrotransposition of Alu elements, while the most frequently affected genes with unique RE insertions were *BRCA2* (45.9%) and *ATM* (16.2%) (Qian et al., 2017). Mechanistically, there is no reason for the observed predominance of *BRCA2* in RE events.

A hereditary pan-cancer gene panel survey of 376,159 individuals in the US revealed 3,461 LGRs in 27 genes (Mancini-DiNardo et al., 2019). In general, SVs accounted for 7.2% of all pathogenic variants detected. The largest proportion of pathogenic LGRs were identified in *BRCA1* (27.4%), followed by *PMS2* (11.7%), *CHEK2* (11.1%), and *MSH2* (8.9%) (Mancini-DiNardo et al., 2019). In a separate study focusing on point-mutation-negative HNPCC patients, 11% of cases harbored large rearrangements in four predisposition genes (*MLH1*, *MSH2*, *MSH6*, and *PMS2*) among which 29.6% affected the *MSH2* gene (van der Klift et al., 2005). Similarly, 15% of the point mutation-negative patients with classical FAP had a genomic deletion in *APC* (Michils et al., 2005). On the contrary, various studies in different populations focusing on *BRCA1*/2 mutation-negative HBOC patients revealed that large rearrangements of *BRCA1* and *BRCA2* genes contributed to only 2–3% of the cases (Agata et al., 2006; Preisler-Adams et al., 2006; Thomassen et al., 2006).

The proportion of pathogenic SVs in a given locus as a fraction of the clear-cut mutations of the gene shows a different ratio pattern. Table 2 summarizes the reported SV ratios in the most relevant susceptibility genes of various cancer predisposition syndromes. Typically, SVs represent 10% (ranging from 0.1 to 60.7%) of the acknowledged mutations of these genes. The high extreme was detected in the *STK11* gene, where 30–60% of all mutations are CNVs (Aretz et al., 2005; Mancini-DiNardo et al., 2019). The ratios are also high in the case of *MSH2* and *PMS2*, where large deletions account for ∼20 and ∼25% of mutations, respectively (Kohlmann and Gruber, 1993; Mancini-DiNardo et al., 2019). *MUTYH* is the less abundant in SVs with its ratio of 0.1% relative to all mutations of the genes (Mancini-DiNardo et al., 2019). The differences with regard to SV frequencies in different genes are conceivably a function of their genetic surroundings and chromosomal complexity. For example, the higher proportion of Alu repeats may contribute to the higher rate of genomic rearrangements in *MSH2* compared to that of *MLH1* (van der Klift et al., 2005). The prevalence of *BRCA1* rearrangements over *BRCA2* is explained also by the differences in the ratio of intronic Alu repeats between the two genes (Judkins et al., 2012; Sluiter and van Rensburg, 2011). Similarly, *PMS2*, due to its extensive pseudogene regions is an especially good subject for rearrangements through recombinations (Smith et al., 2016).

**TABLE 2:** Relative ratios of germline SVs compared to all mutations of the susceptibility gene in various cancer syndromes.

Gene	Syndrome	Ratio of SVs in all mutations of the gene	References
STK11	Juvenile polyposis syndrome	60.7%	Mancini-DiNardo et al., 2019
		30%	Borun et al., 2015
SMAD4		10%	Calva-Cerqueira et al., 2009
BMPR1A		10%	Calva-Cerqueira et al., 2009
SMAD4 & BMPR1A		30%	Aretz et al., 2005

APC	Familial adenomatous polyposis	6%	Kerr et al., 2013
		8.3%	Mancini-DiNardo et al., 2019
MUTYH		0.1%	Mancini-DiNardo et al., 2019

MLH1	Hereditary non-polyposis colorectal cancer syndrome	10%	Smith et al., 2016
MSH2		24%	Smith et al., 2016
MSH6		2.7%	Mancini-DiNardo et al., 2019
PMS2		25%	Mancini-DiNardo et al., 2019
		21%	Senter et al., 2008
		37%	Vaughn et al., 2010

PTCH	Gorlin syndrome	15%	Smith et al., 2016

VHL	Von Hippel-Lindau disease	16.6%	Smith et al., 2016
		25%	Maher et al., 1996

NF1	Neurofibromatosis type 1	12%	Smith et al., 2016

NF2	Neurofibromatosis type 2	20%	Smith et al., 2016

MEN1	Multiple endocrine neoplasia type 1 syndrome	12%	Pardi et al., 2017

CHEK2	Hereditary breast and ovarian cancer syndrome	14%	Nizic-Kos et al., 2020
		15.26%	Kleiblova et al., 2019
PALB2		9.6%	Mancini-DiNardo et al., 2019
		18%	Janatova et al., 2013
RAD51C		21%	Mancini-DiNardo et al., 2019
BARD1		10.2%	Mancini-DiNardo et al., 2019
BRIP1		4.7%	Mancini-DiNardo et al., 2019

ATM	Ataxia telangectasia	5.8%	Mancini-DiNardo et al., 2019

CDH1	Hereditary diffuse castric cancer	14.4%	Mancini-DiNardo et al., 2019
		16.7%	Molinaro et al., 2014

TP53	Li-Fraumeni syndrome	10%	Smith et al., 2016

**FIGURE 2:**
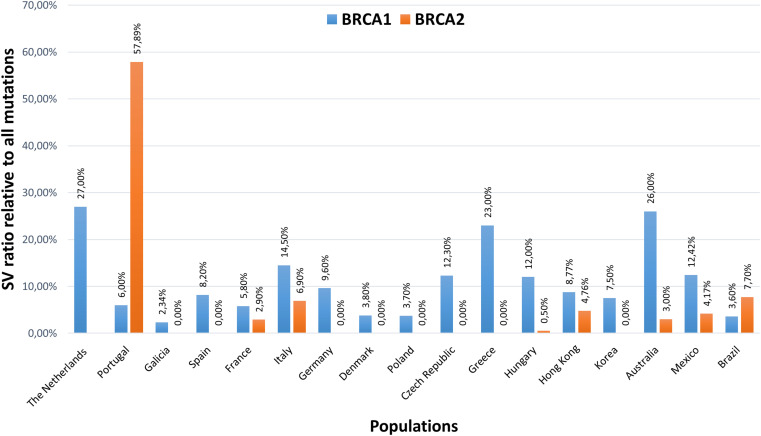
Structural variation ratios of *BRCA1* and *BRCA2* genes relative to all pathogenic mutations detected in HBOC probands of various ethnicities. The Netherlands (Hogervorst et al., 2003), Portugal (Peixoto et al., 2011), Galicia (Fachal et al., 2014), Spain (de la Hoya et al., 2006), France (Caux-Moncoutier et al., 2011), Italy (Concolino et al., 2018), Germany (Engert et al., 2008), Denmark (Thomassen et al., 2006), Poland (Rudnicka et al., 2013), Czech Republic (Ticha et al., 2010), Greece (Armaou et al., 2009), Hungary (Bozsik et al., 2020), Hong Kong (Kwong et al., 2015), Korea (Seong et al., 2014), Australia (James et al., 2015), Mexico (Lopez-Urrutia et al., 2019), Brazil (Palmero et al., 2018).

CNVs in *BRCA1* and *BRCA2* genes, being the most penetrant HBOC predisposition loci, are extensively studied in various populations worldwide. To date, more than 100 LGRs have been characterized in *BRCA1*, whereas much fewer have been characterized in *BRCA2* (Sluiter and van Rensburg, 2011). The CNV ratios of *BRCA1* and *BRCA2* genes relative to all pathogenic mutations detected in HBOC probands of various ethnicities are visualized on the histogram in Figure 2. On average, CNVs account for 10% of all pathogenic mutations of the *BRCA1* gene; the differences in ratios in various ethnicities are mainly attributed to founder mutations. *BRCA2* locus has only low contribution (<0.5%) to CNVs (Hogervorst et al., 2003; de la Hoya et al., 2006; Thomassen et al., 2006; Engert et al., 2008; Ticha et al., 2010; Fachal et al., 2014). A remarkable percentage of *BRCA2* large rearrangements have only been detected in Portugal, where the c.156_157insAlu founder mutation constitutes the bulk of the cases (Machado et al., 2007). An elevated ratio of *BRCA2* CNVs is also observed in male breast cancer patients (Tournier et al., 2004).

Additional association studies are seeking to identify further pathogenic CNVs in breast cancer contributing to the disease phenotype. Kumaran et al. (2017), identified 200 common germline CNVs associated with breast cancer in a whole-genome sequencing study of 422 breast cancer cases and 348 controls (Kumaran et al., 2017). Moreover, they also confirmed, that germline CNVs conferred dosage effects on gene expression in breast tissue (Kumaran et al., 2017). Similarly, another study of genome-wide germline CNVs identified 275 unique rearrangements that potentially contribute to breast cancer initiation and/or progression (Masson et al., 2014).

## Summary

Within germline SVs in CPGs for hereditary cancer syndromes, copy number changes are the prevailing alterations. The predominant CNVs are deletions, affecting various portions of the genes. The main structural source of these deletions is intronic Alu sequences. Double-strand break repairs and additional molecular mechanisms harness these sequence homologies and may result in copy changes through ectopic alignments. Studies conducted in different populations confirmed that SVs generally account for 7–10% of all mutations in CPGs, thus their contribution of mutational burden is significant. Precise detection of these types of alterations is essential to provide an optimal genetic diagnosis. Differences in neighboring genetic architecture, as well as various applied detection techniques, may contribute to the wide range of variations in the exact ratios of pathogenic CNVs compared to point mutations (Mancini-DiNardo et al., 2019) [etc.].

The association with the clinico-pathological phenotype is straightforward in the majority of germline SVs, however, in a few cases, and especially within some duplications, pathogenic effects cannot be addressed unambiguously. Moreover, several studies proposed that copy number changes, although larger in size, do not necessarily associate with a more severe pathological phenotype than smaller-scale indels (Walsh et al., 2006; Rhees et al., 2014; Smith et al., 2016; Bozsik et al., 2020). On the other hand, extensive rearrangements affecting the whole gene together with several neighboring genes may elicit a complex phenotype due to the putative interfering effects of the respective proto-oncogenes and tumor suppressors (Delnatte et al., 2006; Smith et al., 2016). However, since the number of cases harboring such rearrangements is limited, further studies are needed to ascertain these correlations.

Due to the continuous development of dosage-sensitive detection modes and validations, an increasing number of germline structural rearrangements are being discovered in several CPGs. Note that synthesis of the data highlighted differences regarding structural variation types and frequencies between the studied CPGs. This has raised the possibility, that some rearrangement types, mainly inversions and insertions, may be underrepresented as a consequence of genotyping insufficiency, and a significant portion of heritability may remain unexplained with current genotyping assays. For example, in NGS sequencing results spurious deletions, not validated as real copy number losses may be a consequence of allelic dropout or failed alignment of the reads due to possible breakpoints of other types of rearrangements. Genotyping techniques that also enable sequencing of introns are preferred since the majority of rearrangement breakpoints reside in these regions. Equally important, several deletions affecting more genes may manifest in a multilocus phenotype, modulating the typical symptoms of diseases. Therefore, careful evaluation of the syndromic spectrum is warranted for determining the genotyping eligibility criteria.

## Databases

The following curated databases register the detected SVs: InSight LOVD for gastrointestinal hereditary tumors (http://insight-database.org). Breast-and ovarian cancer: ENIGMA, BRCA Exchange (https://brcaexchange.org/variants) and LOVD Fanconi anemia mutation database (https://databases.lovd.nl/shared/genes/BRCA1). General: Database of Genomic Variants (http://dgv.tcag.ca/dgv/app/home). Human Gene Mutation Database (http://www.hgmd.cf.ac.uk/ac/index.php).

## Author Contributions

AB, TP, VG, and AP wrote and carried out the original draft preparation. TP, JP, HB, and AP reviewed and edited the manuscript. AP carried out the funding acquisition. All authors have read and agreed to the published version of the manuscript.

## Conflict of Interest

The authors declare that the research was conducted in the absence of any commercial or financial relationships that could be construed as a potential conflict of interest.

## Footnotes

1 www.mlpa.com

## Abbreviations

SV: structural variation
CNV: copy number variation
LGR: large genomic rearrangement
CPG: cancer predisposition gene
HBOC: hereditary breast- and ovarian cancer
FAP: familial adenomatous polyposis
HNPCC: hereditary non-polyposis colorectal cancer
